# An Overview of Scaffold Design and Fabrication Technology for Engineered Knee Meniscus

**DOI:** 10.3390/ma10010029

**Published:** 2017-01-03

**Authors:** Jie Sun, Sanjairaj Vijayavenkataraman, Hang Liu

**Affiliations:** 1 Department of Industrial Design, Xi’an Jiaotong-Liverpool University, Suzhou 215123, China; 2 National University of Singapore (Suzhou) Research Insititute, Suzhou 215123, China; Hang.Liu12@student.xjtlu.edu.cn; 3 Department of Mechanical Engineering, National University of Singapore, Singapore 117576, Singapore; vijayavenkataraman@u.nus.edu

**Keywords:** meniscal scaffold, additive manufacturing, scaffold design and fabrication

## Abstract

Current surgical treatments for meniscal tears suffer from subsequent degeneration of knee joints, limited donor organs and inconsistent post-treatment results. Three clinical scaffolds (Menaflex CMI, Actifit^®^ scaffold and NUsurface^®^ Meniscus Implant) are available on the market, but additional data are needed to properly evaluate their safety and effectiveness. Thus, many scaffold-based research activities have been done to develop new materials, structures and fabrication technologies to mimic native meniscus for cell attachment and subsequent tissue development, and restore functionalities of injured meniscus for long-term effects. This study begins with a synopsis of relevant structural features of meniscus and goes on to describe the critical considerations. Promising advances made in the field of meniscal scaffolding technology, in terms of biocompatible materials, fabrication methods, structure design and their impact on mechanical and biological properties are discussed in detail. Among all the scaffolding technologies, additive manufacturing (AM) is very promising because of its ability to precisely control fiber diameter, orientation, and pore network micro-architecture to mimic the native meniscus microenvironment.

## 1. Introduction

### 1.1. Meniscus

**Figure 1:**
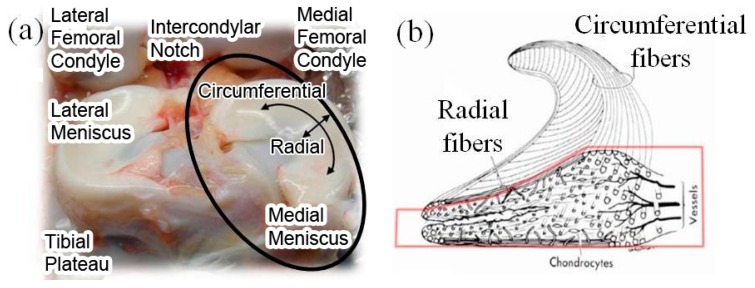
(**a**) Anatomical photograph of meniscus; (**b**) internal microstructure at cross-section. Figure 1 illustration of Human Knee Meniscus (adapted from [1,2]).

Meniscus provides physical protection to knee cartilage by transmitting loads through the joint, distributing high peak stresses on the underlying surfaces, providing shock absorption. The internal microstructure of a meniscus consists of circumferentially oriented collagen fibers and radial collagen fibers, as shown in Figure 1 [1]. The surface is covered by a meshwork of thin fibers of about 35 nm diameter. The radially oriented fibers act as crosslinkers to prevent the longitudinal splitting of circumferential fibers. The diameter of radial and circumferential fibers is about 120 nm, and these fibers form oriented bundles with a diameter of 20–50 µm. Due to the orientated fiber bundle arrangement, the meniscus presents high anisotropies, i.e., different properties in different directions. For the medial meniscus highlighted in Figure 1, its tensile strength is about 50–300 MPa along the circumferential direction and 3–70 MPa along the radial direction [1].

The meniscus consists of three parts: the outer vascular/neural area with 80% collagen type I (dry weight), the inner entirely avascular/aneural area with 70% collagen content (both type II and type I) (dry weight), and the junctional area between the two. Meniscal tears are the most common intra-articular injuries in the knee. From the PearlDiver database which represents approximately 9% of the US population under 65 years, a total of 387,833 meniscectomies and 23,640 meniscus repairs were reported between 2005 and 2011 in the US [3]. Meniscus repair over resection, when feasible, should be strongly considered in an effort to preserve meniscus integrity and function [4]. There are two surgical treatments commonly used to treat meniscal injuries, namely, meniscectomy allografts and sutures. Meniscectomy is the surgical removal of all or part of an injured meniscus and can be classified respectively as total meniscectomy and partial meniscectomy [5]. However, post-treatment studies prove that meniscectomy increases the risk of radiographic degenerative changes, associated with severe pain and dysfunction, which is expected to progress over a long-term period resulting in decreased patient satisfaction [6,7]. Meniscal transplantation (allografts) has been proposed as a possible solution to these problems. Although evidence suggests that meniscal allograft transplantation provides improvement in the short and intermediate term with regard to pain and level of function for daily activities, other aspects like limited donors, graft selection, sizing, sterilization and preservation remain controversial areas; also, the protective effect of meniscal allograft transplantation against progression of degenerative joint disease remains unproven [8,9]. Due to the above limitations of current surgical treatments, there is a need for a more consistent approach to restore the functionalities of the injured meniscus, which resulted in the development of scaffold-based tissue regeneration.

### 1.2. Meniscal Scaffold

In regenerative medicine, scaffolds are used as a temporal template to facilitate host tissue integration upon implantation, for new tissue formation and remodeling. From a structural or mechanical perspective, meniscal scaffolds should (1) provide appropriate biomechanical functions after implantation to shield cells from damaging compressive or tensile forces; (2) maintain their shape integrity (without shrinkage, etc.), mechanical stability and strength on the defect area until enough host tissue has been regenerated; (3) produce mechanical stimuli to promote tissue regeneration [10].

From a biological perspective, meniscal scaffolds should: (1) possess appropriate/acceptable biocompatibility and nontoxicity profiles (due to direct contact with living tissues); (2) provide suitable surface properties (e.g., hydrophilicity) and mechanical properties (e.g., stiffness) for cell attachment, proliferation and differentiation, and facilitate cell–cell contact and cell migration; (3) have sufficient porosity for the cell media and growth factors to permeate and reach the cells in all the layers of the scaffold; and, (4) serve as a delivery vehicle or reservoir for exogenous growth-stimulating signals such as growth factors. In addition, the degradation rate of scaffolds should match the rate of specific tissue regeneration so that they can disappear completely once the tissues are repaired. Degradation byproducts from scaffolds should also be biocompatible and removable with minimal immune or inflammatory responses and without further surgeries [10].

Three types of biodegradable and biocompatible scaffolds are available on the market to reconstruct the segmental meniscus defects: Menaflex CMI from ReGen Biologics, Inc. (ReGen Biologics, Inc., Cary, NC, USA), Actifit^®^ scaffold from Orteq Ltd. (Orteq Ltd., London, UK), and NUsurface^®^ Meniscus Implant from Active Implants (Active Implants, LLC., Memphis, TN, USA). Menaflex CMI and Actifit^®^ scaffold are partial meniscal substitutes with equivalents in histological, radiological, and clinical evaluations [11]. They have received the Conformité Européenne (CE) mark in Europe, whereas the US Food and Drug Administration (FDA) believes that additional data are needed to confirm their efficacy on chondral degradation and prevention of osteoarthritis development [11]. NUsurface^®^ Meniscus Implant is the first total meniscal substitute, and has been used in Europe under CE Mark since 2008 and in Israel since 2011 [12]. The clinical results in the short term seem to be as promising. About 130 middle-aged patients have been treated, and significant pain relief was reported after post-operation 12 months [13].

Although these scaffolds are designed to mimic the function of the natural meniscus or stimulate the growth of new tissues, they need to be improved in terms of structure design, material design and so on.

### 1.3. Cell Sources for Meniscal Scaffold

Both mesenchymal stem cells (MSCs) and costal chondrocytes (CC) have been used in the study of meniscal scaffolds. MSCs which can be readily harvested from numerous adult tissues, can differentiate into cell types indigenous to bone, cartilage, muscle, tendon, ligament, and connective tissues [14]. MSCs, after being embedded in the scaffolds, can produce new matrix to repair defects, including type I collagen sponges [15], decellularized meniscus [16] and hyaluronan/gelatin composites [17]. CC, a clinically compliant cell source, can be harvested abundantly with little donor site morbidity. They display a high synthetic activity after in vitro expansion and generate large quantities of fibrocartilaginous matrix with glycosaminoglycans (GAGs), collagen type II and type I [18]. Co-culturing CC with other cell types can create a spectrum of fibrocartilaginous engineered tissues.

Researchers around the world are working on various tenets of meniscal scaffolds like material selection, cell sources, novel fabrication technologies and optimal biomimic structure design. Such information is scattered in various publications and websites with different technical focuses. The objective of this paper is to collate, categorize, analyze and summarize the information pertaining to meniscal scaffold design, fabrication and mechanical properties, as well as to provide a critical insight into the direction of their future development.

Four types of scaffold materials are reviewed in Section 2, including tissue-derived materials, Extra Cellular Matrix (ECM) components, synthetic polymers and hydrogels. Section 3 reviews the commercially available meniscal scaffolds used in clinical settings. Section 4 discusses general requirements for meniscal scaffolds, sponge and fibrous scaffold structural characteristics, and their application in meniscal scaffold design. With varied fiber diameter, pore size, internal configuration, diverse meniscal structures with circumferential pattern have been designed to mimic native fiber direction variation in the meniscus. The impacts from scaffold structure design on mechanical properties are also discussed. Scaffold fabrication technologies are reviewed in Section 5, including sponge scaffold fabrication, non-woven fibrous scaffold and woven scaffold fabrication. Desirable scaffold properties under both acellular condition and cell culture condition are discussed in Section 6.

## 2. Materials for Meniscal Scaffold and Cell Source

Four types of materials are widely used for meniscal scaffold fabrication.

### 2.1. Tissue-Derived Materials

Tissue-derived materials include periosteal tissue [19], small intestine submucosa (SIS) [20], acellular porcine meniscal tissue [21] and decellularized tissue/ECM [22]. These highly biocompatible materials can be trimmed to match the size and shape of meniscal defects. The acquired scaffolds can be tissue engineered to a functional tissue/organ by culturing stem cells, allowing them to differentiate and develop into desired tissues. The hypothesis of using such materials is that they constitute a natural environment for cell seeding, migration, and ECM deposition. Though geometric fidelity and bioactivity of these scaffolds can be high, they must be procured from natural tissue, and thus supply is problematic. In addition, some decellularization and processing protocols compromise the mechanical integrity of these tissues [23]. Furthermore, the regenerated tissues also lack the required mechanical strength. SIS is a processed whole tissue and several investigations have demonstrated the high bioactivity of such scaffolds. In one study, canine chondrocytes are seeded in SIS and polylactic co-glycolic acid (PLGA) scaffolds and implanted in athymic mice; it was found that sulfated GAG and hydroxyproline content were higher in SIS scaffolds than that in PLGA scaffold [24]. Another study demonstrated that SIS favors retention, infiltration and viability of canine meniscal cells than other dermis isolates (human, fetal bovine, and crosslinked porcine) [25]. Though processed whole tissues such as SIS display significant bioactivity and have been seen to induce some tissue regeneration, the resulting tissue mechanics may be insufficient, which subsequently compromises knee function. Another challenge associated with tissue-derived scaffolds like SIS is difficulty in achieving desired pore sizes. Studies of native menisci have deemed pore sizes of 100–150 microns as appropriate for meniscus cells [26], yet cell infiltration can be highly variant through the depths of both whole processed tissue (SIS, dermis, etc.) [25] and decellularized meniscus [27], likely due to the dense matrix present even after processing. Though there are many advantages in using tissue-derived materials for meniscal repair like high bioactivity and natural environment for cells, they suffer from several drawbacks. Two main drawbacks are the insufficient mechanical properties and supply deficit as it must be procured from natural tissue.

### 2.2. Extra Cellular Matrix (ECM) Components

ECM components are naturally derived, such as collagen, proteoglycans and elastin molecules [28]. Scientists from Regen Biologics Inc. applied a pressure heat molding approach to shape a bovine Achilles tendon into Menaflex collagen meniscal implant (Menaflex CMI). Once implanted, about 75% of the missing meniscal tissue grew back, and fibrocartilaginous tissues similar to normal meniscal tissue were observed [4]. A ten-year follow-up clinical study showed pain relief and functional improvement on knee joints [10,29,30]. The meniscal allografts were observed to shrink [31] and undergo collagen remodeling upon transplantation, which may compromise their mechanical strength. Also, the true efficacy of the implant for prevention of knee osteoarthritis is not clear due to relatively few clinical studies [32].

### 2.3. Synthetic Polymers

Synthetic polymers used for meniscal scaffolds include polyurethane (PU), polyglycolic acid (PGA), polylactic co-glycolic acid (PLGA) and polycaprolactone (PCL). They can be easily shaped to scaffolds with varied pore size, fiber dimension, geometry, porosity, better mechanical properties and an adjustable degradation rate [33].

However, their hydrophobic properties, aseptic inflammation, immune responses and side effects from degradation byproducts limit their applications. For example, diisocyanate from degradation of PU is toxic. The degradation of PLGA generates acidic byproducts, which cause inflammatory responses and damage local tissues [34].

Multiple synthetic polymers are mixed together in suitable proportions to enhance the cell-adhesive properties and lower foreign-body reaction. When polyethylene terephthalate (PET) was added into hyaluronic acid/PCL scaffolds, more type II collagen was observed [35]. An Actifit^®^ scaffold (20% PU and 80% PCL) was implanted in a dog and, after about two years, viable living “meniscus-like” tissue was observed, i.e., a fibrous tissue containing type I collagen and fibrocartilage-like tissue containing proteoglycans and type II collagen [36]. Canine studies revealed full integration, with evidence of meniscus-like tissue ingrowth and only minimal immunological response [37]. Further studies confirmed no deleterious effects on the articular cartilage and a friction coefficient similar to that of the native meniscus 3 months after implanting [31,38,39].

Biopolymers, such as silk fibroin (SF) derived from silkworms, have also been applied to meniscus repair applications due to better biocompatibility, slow degradability and relatively better mechanical properties.

Mandal et al. [40] used SF to recapitulate a three-layer meniscal scaffold, where the first two layers were fabricated by salt leaching and the third layer by freeze-drying. An increased production of sulfated GAGs and a colonization of ECM similar to native tissues were observed after 28 days of chondrocyte culture. Yan et al. [41] reported the compressive modulus of 16% SF scaffolds was comparable to that of natural meniscus. Gruchenburg et al. [42] reported a higher equilibrium modulus in the durability study of SF scaffolds using 17 sheep. The disadvantages of SF scaffolds include foreign body response, low porosity and interconnectivity.

### 2.4. Hydrogels

Hydrogels are semi-liquid hydrophilic colloids constructed by a network of crosslinked natural or synthetic polymer chains. They can be easily cast into desired shapes and evenly mixed with seed cells and growth factors. The physical properties of hydrogels are determined by their water content. Higher porosity benefits cell diffusion, but it has poor mechanical properties. Thus, double-network (DN) hydrogels were developed, which can offer excellent mechanical properties—even with the water content exceeding 90%—and a dynamic stiffness value comparable to that of the swine meniscus [43]. Crosslinking methods involved in DN hydrogel preparation may be weak mechanical properties and speed degradation during implantation. Some crosslinking agents such as glutaraldehyde enhance the biological stability, but suppress the implants’ immunogenicity and lead to over-crosslinking and cytotoxicity [44].

A table of comparison of the above four scaffold types against three criteria (mechanics, bioactivity, logistics) is shown in Table 1. “Mechanics” refers to mechanical properties, geometry, anisotropy, and lubrication; “bioactivity” includes cell phenotype, ECM synthesis, immunogenicity, and potential for host tissue integration; and logistics refers to supply, material processability, sterilization, and ease of surgical implantation. Comparing all the above four types of scaffolds, none of them was superior to the others in terms of biological and biomechanical properties. A possible solution is to combine multiple materials and develop hybrid materials or structure with enhanced properties that facilitate tissue regeneration.

## 3. Scaffold Structure Characteristics and Meniscal Scaffold Structure Design

### 3.1. General Requirements for Meniscal Scaffolds

**Figure 2:**
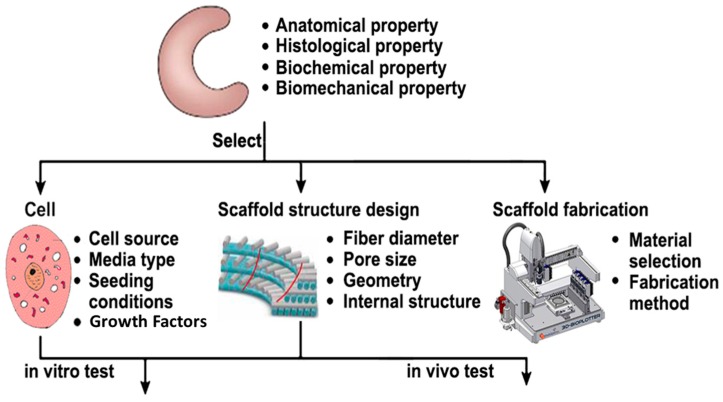
Scaffold-based approach for meniscus regeneration.

As shown in Figure 2, the current study on meniscal scaffolds starts with anatomical, histological, biochemical and biomechanical properties of native meniscus. The three most important focus areas are cell source, scaffold structure design and fabrication technology. The scaffolds seeded with living cells will be implanted into animal models for in vivo testing or cultivated using a bioreactor in vitro (providing mechanical and biochemical cell-signaling stimuli) to attain some degree of functionality before implanting into animal models.

Structural and architectural properties of the scaffolds play an important role in determining the overall scaffold mechanical properties, in additional to the inherent material strength. They can be obtained by patients’ computer tomography (CT) or magnetic resonance imaging (MRI) datasets. The datasets are processed with CAD/CAM modelling software and the final model is transferred to a system for fabrication. CT datasets performed better than MRI when reconstructed tissues had an intrinsic degree of opacity [52].

The required biological properties of scaffolds can be achieved by proper selection of scaffold materials, bioactive agents, cell source and architecture. The most commonly used meniscal scaffolds are sponge or fibrous structure. Both of them can achieve higher porosities and exhibit mechanical properties (compression Young’s modulus) similar to that of natural cartilage, but the dynamic stiffness of fibrous scaffolds is higher than that of sponge scaffolds for the same material [53].

### 3.2. Sponge Scaffold Structure Characteristics

Sponge scaffold structure is largely controlled by the fabrication process rather than design parameters [54]. This structure contains random, smaller pores and has a higher chance of pore blockage than fibrous structures, resulting in poor nutrient diffusion. The overall porosity and level of pore connectivity can be regulated by the ratio of polymer/porogen, the property of porogens and the size of porogen particles. Sponge scaffolds with macropores ranging from 300 to 500 μm have varying mechanical properties and those with lower porosity have better mechanical performance in compression and stress tests [55].

Welsing et al. [56] conducted a two-year follow-up study in 13 dogs using the sponge meniscus scaffolds, and reported that the new generated tissues lacked a specific structural organization and a fibrocartilage phenotype, after both 6 and 24 months. Cell death was observed in the central region of the scaffolds, and articular cartilage degeneration was observed similar to the knees that underwent meniscectomy. The sponge scaffolds’ isotropic properties cannot satisfy the load-bearing requirements and adequately stimulate tissue regeneration, even though some promising results were achieved in in vitro tests [55].

### 3.3. Fibrous Scaffold Structure Characteristics

Fibrous scaffolds are architecturally characterized by micro features such as fiber diameter, pore size and internal configuration of deposited fibers, which determines the porosity and mechanical properties of fibrous scaffolds. The extrinsic stiffness of scaffolds with the same micro features (porosity and pore architecture) but dissimilar geometries resulted in different mechanical responses [52]. Mechanics, bioactivity and logistics are the three important criteria for an ideal meniscus construct. Since heterogeneous loading of the meniscus occurs every day in vivo, appropriate mechanical properties, tissue anisotropy, geometry, and lubrication are requirements of the mechanics criterion. Any implanted meniscus construct will also need to display sufficient bioactivity. This means maintenance of cell phenotype, induction of ECM synthesis, lack of immunogenicity, and capacity for host-tissue integration. Finally, the logistics of a successful construct must not be unwieldy: supply, processability, sterilization, and eventual surgical implantation must all be practical [23].

Fiber diameter is a crucial scaffold design parameter. Scaffolds with smaller fiber diameter can lead to higher porosity and lower tensile modulus. Cell adhesion and growth kinetics are also significantly affected by fiber diameter [33]. To promote specific effects upon cell behavior, bimodal scaffolds (one structure with both microfiber and nanofiber in one region) and biphasic scaffolds (one structure with separated microfiber and nanofiber regions) are fabricated to mimic microenvironments encountered in vivo [33].

Pore size should vary for different kinds of cells and tissues, since appropriate pore size provides a better tissue growth environment. For example, 20–125 μm for skin regeneration [57], 200–350 μm for osteoconduction [58] and 100–400 μm for bone regeneration [59]. Large pore size can provide effective transport of biofactors/growth factors, nutrient supply and metabolic waste removal, though a low intracellular signaling between cells [60]. A denser scaffold with thicker fibers or smaller pore size could provide better mechanical functions such as tensile, compressive or yield properties, but also poor mass transport characteristics [61]. For bigger pore size, scaffold compression modulus increased dramatically after cell culture [62].

For the same material, some technologies can produce scaffolds with higher porosity than others. PCL scaffolds fabricated by fused deposition modeling (FDM) [63] can achieve a higher porosity (ranging from 48% to 77%) than that of selective laser sintering (37% to 55%) [64].

Cells are capable of sensing mechanical rigidity in their surroundings at different scales [65]. When the stiffness of scaffolds matches the native environment, it enhances cell differentiation and produces higher ECM. Except bulk material properties, the mechanical properties of scaffolds are greatly determined by structural arrangement, such as pore shape and internal structure. Scaffolds with spherical pores can produce more robust ECM and better mechanical properties (stiffness and nonlinearity) than those with cubical pores [66].

Interconnected structures with open pores can facilitate homogeneous cell seeding and better nutrient dispersion throughout the construct, especially for vascular, bone and other soft tissue-related applications.

### 3.4. Fibrous Scaffold Structure Design with Circumferential Pattern

In the meniscus, dense collagen bundles are locally aligned and this alignment changes gradually along the height. As a result, a circumferential pattern is formed aiding better load transmission across the knee joint. Meniscal scaffold structure should mimic such directional variation so as to provide the necessary circumferential tensile strength. This can be done by modifying the fiber deposition patterns from a homogeneously perpendicular architecture to a more radially oriented configuration.

Moroni et al. [52] used Bioplotter (Envisiontec GmbH, Gladbeck, Germany) to manipulate fiber deposition patterns. Varied angles (0°/45°/90°/135°) were chosen to deposit fiber structure at the bottom and top 1 mm while 0°/90° angle were chosen to be deposited in the middle part. It was reported that the extrinsic stiffness (overall mechanical response) and equilibrium modulus of this porcine meniscus scaffold could be modulated by structural parameters such as pore size and fiber orientation.

**Figure 3:**
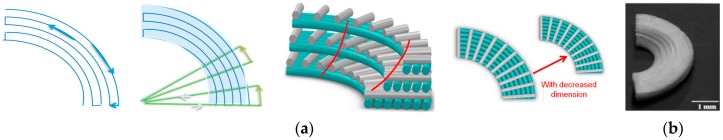
(**a**) Fabrication pathway; (**b**) 3D wedge-shaped meniscal scaffold (adapted from [68]).

Balint et al. [67] fabricated a five-layer fiber-reinforced meniscal scaffold with a wedge-shaped cross-section. Figure 3 shows the specific pattern at each layer, the combined pattern model and a fabricated sample. The polymer fiber was melt-extruded with an average diameter of 80 µm and wrapped in a 3D quasi-circumferential pattern. Each layer was extended to an anchor to reinforce the main body, which shares axial loads across the knee joint by generating tensile forces. This design was benchmarked with the normal ovine meniscus using circumferential tensile test, and it was reported that this scaffold can mimic the tensile and hoop stress behavior of normal meniscal tissue under compressive loading. This study only focusses on the design of biomimetic meniscal scaffold and its mechanical characterization, no cell culture studies were performed on the fabricated scaffolds.

Our group at National University of Singapore chose five independent parameters (outer radius (R), number of circumferential fibers (N), gap between two circumferential fibers (G), number of radial fibers (n), and angle between the adjacent radial fibers (θ)) to design meniscal scaffolds using Electrohydrodynamic-jetting (EHD-jetting) [68]. As depicted in Figure 3a, the fabrication starts with printing the circumferential fibers first followed by the radial fibers. The number of circumferential fibers were reduced progressively after every 20 layers to form a wedge shape, as shown in the fabricated 300-layer scaffold in Figure 3b. The fabricated sample could withstand greater tensile stress along the circumferential direction and sustain larger deformation before yield, i.e., good capacity to absorb loads and disperse pressures in the loop.

In testing the functionalities of various scaffold structure designs, compression and tensile properties have received more attention. The 3D-woven scaffolds with fiber-reinforced, gradient structure [54] or oriented structure [68,69] demonstrate considerable improvements on anisotropic mechanical properties, as well as high tensile strength and stiffness. Oriented fibrous scaffolds have mechanical and cellular properties, similar to that of the native meniscus as it is more biomimetic than scaffolds with non-oriented and random fibers. Gradient structures in woven fibrous scaffolds with varying porosities allow tailoring both mechanical and architectural properties with minimal compromise. To optimize the meniscal scaffold structure design, it is important to investigate various factors which influence the biological properties. Currently, these designs are evaluated by trial and error method, i.e., collecting and analyzing data in vitro and/or in vivo and working on design improvement. The exact mechanisms that affect structure/composition are not fully understood. Although computer simulation was introduced to estimate mechanical properties of different scaffold structures [52], the progress is very limited. To date, we do not have powerful quantitative methods to analyze structural properties from biomechanics and biological perspectives and evaluate scaffolds under mechanical loadings for specific applications.

## 4. Meniscal Scaffold Fabrication Technologies

Meniscal scaffold fabrication can be as challenging as the scaffold design, with the consideration of bulk material properties, structure design and functional requirements. The fabrication process for either sponge or fibrous structure scaffolds should be reproducible and reliable.

### 4.1. Sponge Scaffold Fabrication Technologies

Traditional technologies like particulate leaching [70], gas foaming [71,72], freeze drying [73] and phase separation techniques [74,75] are used to fabricate sponge structure scaffolds. In these processes, biomaterials and porogens are mixed together and then they are casted or extruded to fabricate scaffolds. After porogens are removed using sublimation, evaporation and melting, porous structures are created in the scaffolds. Biomaterials used in these processes can be either solids or dissolved in solvents and porogens could be gases such as carbon dioxide, liquids such as water or solids such as paraffin.

A thermal-induced phase separation process [75] was used to produce anisotropic scaffolds with channels, where the frozen polymers were lyophilized overnight to remove water and dioxane crystals. Tissue infiltration and collagen alignment along the channels were observed in such scaffolds, similar to the vascularized zone in the native meniscus. Esposito et al. [76] integrated solvent casting and particulate leaching to fabricate rabbit menisci scaffolds using PLDLA/PCL-T (90/10) solutions. They reported that these scaffolds adapted well to surrounding tissues, without apparent rejection, infection, or chronic inflammatory response. Fibrocartilaginous tissue with mature collagen fibers was observed after implanting 24 weeks. Chiari et al. [77] used salts to increase the porosity of a meniscus substitute in sheep for better intercommunication among pores. They reported that a large number of blood vessels were developed, growing from the superficial to the central areas of the scaffold structure [77].

The sponge scaffold fabrication techniques are either based on physical or chemical treatment, with associated pressure and/or temperature change. Such a harsh environment makes pre-seed cells impossible to survive or function properly. The residues from the organic solution used during the fabrication process are more or less detrimental for cells and bioactive molecules. The scaffold properties are isotropic and their internal structures are largely controlled by the fabrication process rather than the parameters. In other words, it is very hard to control pore size and geometry, pore distribution, interconnectivity, and porosity. Thus, they suffer more from pore blockage (filtration effect) than the fibrous structures.

### 4.2. Non-Woven Fibrous Scaffold Fabrication with Orientation Control

A fibrous scaffold structure consists of individual fibers either in woven or non-woven patterns with variable pore size, shape and fiber diameter. The advantages of such a structure include a large surface area for cell attachment and rapid diffusion of nutrients in favor of cell survival and growth. Electrospinning and additive manufacturing (AM) are the technologies that are being used for non-woven and woven fibrous scaffold fabrication individually.

#### 4.2.1. Electrospinning Technology in Fibrous Scaffold Fabrication

Electrospinning technology is increasingly being used to process synthetic and natural polymers for fabricating fibrous scaffolds. By controlling the various process parameters like the solution conditions (concentration and solvent type), process conditions (distance between tip and plate, strength of electric field, and dimensions of nozzle), and collection methods (plate versus rotating mandrel and speed of collection), this technique can produce ultrafine disordered fibers from several micrometers to a few nanometers, similar to the size of collagen fibers in the meniscus [78]. Despite numerous benefits, densely packed structures with randomly oriented electrospun fibers limit this technology’s capability to control the fabricated scaffolds’ structure and porosity.

#### 4.2.2. Rotating Devices to Align Electrospun Fibers

Collagen fibers or uniaxial fiber bundles in the meniscus are organized either in parallel or in perpendicular direction to the surface. To incorporate such spatial cues into meniscal scaffolds, rotating dynamic collectors are integrated into electrospinning set up to align fibers, which could be a disc collector [79], rotating drum/mandrel [80] or a rotating tube with knife-edged electrodes [81].

Baker et al. [13] rotated a drum/mandrel to align electrospun fibers in a 2D mesh and then constructed 3D wedge shapes via lamellar folding and spot-welding of this mesh. Ionescu and Mauck [82] used a rotating mandrel to control the porosity of nonwoven scaffolds and alignment level; disorganized scaffolds (no prevailing fiber direction) and aligned scaffolds (single predominant fiber direction) were compared. The latter’s mechanical property was seven times higher than that of the former, approaching the native meniscus properties after 10 weeks in vitro. Besides, highly porous electropun scaffolds integrate better with a native tissue and mature to a greater extent.

Fisher et al. [83] rotated a circular surface to produce scaffolds with circumferentially aligned nanofibers. This method produced nanofibrous scaffolds with a spatially varying macroscopic fiber orientation, similar to the meniscus. A similar local morphology was showed after cell seeding, where circumferential cellular alignment was generated with juvenile bovine MSCs.

The above rotating devices can quickly and roughly align fibers in a limited orientation and create meshes/scaffolds with aligned nanofibers. However, it is difficult to use these electrospun fibers to construct scaffolds with greater architectural complexity (e.g., gradient structure, spatially controlled properties). Furthermore, both sponge scaffold and non-woven fibrous scaffold fabrication methods result in random internal structure and vary from part to part and thus lack repeatability.

### 4.3. Additive Manufacturing (AM) in Woven Meniscal Scaffold Fabrication

AM is a computer-controlled solid free-form fabrication technique that can reproduce scaffolds with consistent internal and external architecture. A scaffold model based on medical images (MRI or CT) or customized design, can be physically built layer by layer.

Common AM technologies such as fused deposition modeling (FDM) and precision extrusion deposition (PED), were used to fabricate synthetic material scaffolds with fiber diameter of about 100–500 µm [84,85]. In FDM, the melted semi-solid polymer is extruded, solidifying almost immediately and welding to the previous layers. Schantz et al. [86] used an FDM system (Stratasys, Eden Prairie, MN, USA) to fabricate uniform reproducible scaffold architecture. The fabricated PCL/CaP scaffolds had a fiber diameter above 250 μm with interconnected pore size in the range 300–500 μm.

PED consists of a mini-extruder mounted on a high-precision positioning system. This technique uses granulated materials, which avoid most of the material preparation steps in FDM. Shor et al. [86] used PED to fabricate PCL and composite PCL/hydroxyapatite (HA) tissue scaffolds. Two sets of cylindrical scaffolds were fabricated with pore size of about 450 μm and porosities between 60% and 70%.

Due to the extra-large pore size and large fiber diameter (above 100 micrometer), FDM and PED cannot create scaffolds to benefit cell growth. The nozzle blockage is another problem, especially when extruding viscous materials through a small diameter nozzle. To overcome these bottlenecks, AM needs to be integrated with a high-resolution fiber fabrication technology to advance meniscal scaffold fabrication.

#### 4.3.1. Electrohydrodynamic-Jetting (EHD)-Based AM Platform for Fiber Orientation Control

**Figure 4:**
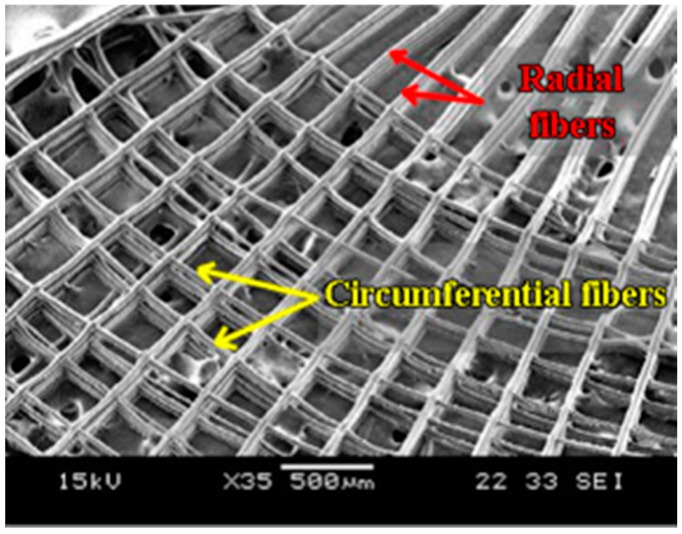
Fabricated meniscal scaffold (adapted from [68]).

To generate micron to nanoscale fibers, EHD-jetting, a near-field electrospinning process, is introduced. This process uses a pneumatic or syringe pump to supply material solution to a nozzle at a constant flow rate, and an applied electric field between the substrate and the nozzle to pull very fine fibers out of the nozzle. This technique is capable of producing micron to nanoscale scaffolds with high-resolution patterns, controlled structure and porosity, optimized material formulations and path planning [87]. When this EHD-jetting is integrated with a three-axis (or four-axis) high-precision motorized stage, the fibers generated can be precisely orientated and woven into wedge-shaped scaffolds with tailored microstructure to mimic the circumferential pattern in the meniscus as shown in Figure 4 [68]. Further examination showed that the organization of actin cytoskeletal network on the EHD-jetting scaffolds was in an orientation manner, with elongated actin filaments along the circumferential and radial fibers. This EHD-jetting based AM platform can reliably produce and precisely control not only the scaffold structures, but also the material distribution.

Most importantly, the fabrication parameters can be optimized and standardized, thus improving the repeatability and reliability of scaffold fabrication. A strategic study of cell and scaffold interaction becomes possible, which is particularly important for scaffolds with specially designed physical or biological features.

An intelligent process monitoring is expected to optimize this fabrication technology through the control of current/voltage amplitude, waveform, distance between the nozzle and the substrate, solution feed rate and so on. It should not only perform real-time adjustment, but also be able to cope with disturbance from unpredictable factors such as nozzle circularity, substrate material impurities, inhomogeneous solution, as well as voltage and pressure fluctuations.

#### 4.3.2. Other AM Techniques for Biomimetic Scaffold Fabrication

The combination of hydrogel and solid polymers can resemble the biphasic nature of meniscus (water and solid) and produce a cell-friendly environment [88]. The current way is to drop hydrogel cell suspension onto the solid scaffolds or seed hydrogel spheroids into the scaffolds. However, they cannot control material distribution over the structure.

To overcome this shortcoming, AM techniques have been used to fabricate hydrogel-based scaffolds with designed patterns and incorporate growth factors, microspheres or nanoparticles into fabrication process. For example, drop-on-demand (DOD) micro-dispensing AM technology, has been used to precisely dispense solid/hydrogel components with customized spatial distribution or varied composition to develop a biomimetic cellular scaffold. Chang et al. [89] used DOD to deposit viscous hydrogels using four types of nozzles, and each print head is unique in the sense of its operation method. Shim et al. [90] employed a multi-head deposition system to dispense synthetic polymer and hydrogel, with varied combinations and position. Schuurman et al. [91] constructed viable hybrid constructs by depositing PCL polymer and cell-laden hydrogels (alginate) layer by layer, through a pneumatic syringe dispenser (hydrogel) and a polymer dispenser. To obtain the desired droplets in DOD micro-dispensing for biomimetic scaffold fabrication, optimal physical design of print-heads and characterizing the operating parameters are a prerequisite. Zhao et al. [92] have demonstrated the feasibility of using electrohydrodynamic printing to pattern microscale liquid PVA lines as well as fluorescent microparticles on transparent ITO-treated glass slides, which could be potentially used for high-resolution hydrogel/cell patterning for the studies of microscale cell-cell interactions or organ printing.

The electrospinning technique has also been integrated into the AM platform to dispense viable cells or collagens in hybrid scaffold fabrication. Lee et al. [93] applied this technique to fabricate hybrid PCL/collagen scaffolds, where a plotting system driven by a three-axis robot stage was used to draw PCL micro-sized strands and an electrospinning system was used to generate layered nano-sized collagen fibers.

Although the above studies are not specifically aimed at meniscus scaffold fabrication, they shed light on the feasibility of using advanced AM techniques to fabricate scaffolds with controlled and designed distribution of cells, hydrogel and bioactive agents.

Common fabrication technologies used in meniscal tissue engineering are tabulated in Table 2, including the pros and cons.

## 5. Scaffold Properties

The performance of scaffolds must be evaluated, not only in the relatively controlled in vitro environment, but also in the context of tissue’s physiological function (e.g., under mechanical stimulation), and ultimately in in vivo conditions. The difference between scaffold degradation behavior in in vitro and in vivo [94] should also be considered, and the latter indicates scaffolds’ actual performance in clinical applications.

### 5.1. Static Mechanical Properties

Meniscus is a C-shaped disc of fibrocartilage with a triangular cross-section, where type I and type II collagen fibers are arranged in a circumferential pattern. To document the differences between native meniscus and meniscal scaffolds, scaffolds are usually tested with respect to one or two mechanical properties at the expense of others. Tensile test and compressive test are the most common ones.

For native meniscus, tensile properties are cross-section and position dependent due to varied fiber densities and orientations, as a result axial compressive force being shared as circumferential tensile load. Tensile test is to quantify the circumferential tensile properties of scaffolds when the knee bears an axial load. Compressive test is to determine the ability of scaffolds on sharing compressive loads via generating tensile loads. Scaffold structure can significantly influence the results from the tensile and compressive tests. For gradient structure scaffold [54] or oriented structure [68], the interaction among different layers results in heterogeneous ECM formation. Thus, mechanical properties (compression modulus) are depth-dependent, compared to homogeneous structure scaffolds.

Scaffolds’ mechanical properties also change with the passage of time because of biomaterial degradation and surrounding ECM formation. The former lowers the mechanical strength of the meniscal scaffold structures and the latter increases it. Overall, the tensile modulus of cell-laden scaffolds is significantly higher than the acellular scaffold [67].

### 5.2. Scaffold Properties under Mechanical Stimulation

Structural and functional properties of scaffolds can be enhanced under controlled mechanical stimulation (dynamic cell culture). This can modulate the spatial heterogeneity of engineered menisci, with higher stiffness in tension and compression [95]. Dynamic compression on micro-channeled scaffolds resulted in aligned cell layers and collagen fibers [96] and hydrostatic pressure combined with growth factor can enhance compressive properties [97]. Other stimulations were also reported to increase ECM production and newly formed tissues’ mechanical properties, such as tension-compression loading [98], perfusion and cyclic compression [99].

### 5.3. Scaffold Properties under Degradation

The scaffold degradation is controlled by material properties and composition, local environment (temperature and pH), structure, and mechanical loading conditions. Even the degradation mechanism has not been quantitatively determined: both pore wall thickness (bulk degradation) and surface area (surface degradation) determine scaffolds’ degradation rate. Scaffolds with higher porosity or smaller pore sizes degrade more slowly than those with lower porosity or larger pore sizes [100]. Highly crosslinked hydrogels (smaller mesh size) exhibit longer degradation time. An ideal degradation rate should be proportional to the tissue-formation rate, until the newly generated tissues function properly.

### 5.4. Challenges

To benchmark the scaffold property tests, the authors have identified three critical issues to be further researched. One is to develop consistent mechanical and biological tests and/or evaluation criteria. Most of the results are based on a variety of testing conditions and criteria. It is very necessary to develop standard evaluation protocols for mechanical and biological assessment. This will be beneficial to compare different scaffold designs and develop better strategies.

Testing results published with in vivo studies are considerably fewer in number than that with in vitro. For the same scaffold, the relationship between in vivo and in vitro performance has become another critical issue to be addressed. Last but not least, scaffolds with excellent mechanical properties cannot guarantee the regenerated tissues will have good mechanical properties. Whether a biomimetic design will actually result in a functional (mechanically and/or biologically) engineered meniscus, is still under research. Further studies are expected to explore the linkage between in vitro and in vivo, and analyze design-based functions.

## 6. Conclusions

This study summarizes the current research activities on meniscal scaffold in terms of materials, fabrication methods, structure design and their influence on mechanical and biological characteristics. Strategic scaffold designs have been developed using biological and structural knowledge from native meniscus and/or its healing process. The current major challenges are still material property and fabrication capabilities, in terms of biochemical composition and biomechanical properties. To restore an intact meniscus with competent functions, researchers should also explore cell-seeding techniques and medical imaging technology. Of all the fabrication technologies, electrohydrodynamic (EHD)-jetting and drop-on-demand (DOD) micro-dispensing techniques based on the additive manufacturing (AM) platform are more promising for producing multi-material scaffolds with solid framework structure or cell–hydrogel formulation with controlled material distribution. They greatly reduce the steps required for scaffold preparation and fabrication, thus accelerating an economical transition to clinical applications. Last but not least, developing mathematical models to simulate the tissue-formation process and the relationship between scaffold properties and design features would benefit scaffold optimization.

## Figures and Tables

**Table 1:** Properties of different types of materials used in engineered meniscal scaffolds.

Materials	Properties					Reference
	Mechanics			Bioactivity	Logistics	
	Mechanical Properties (Elastic Modulus)	Anisotropy	Geometry (Biomimetic)			
Tissue-derived Materials	Periosteal tissue: 8–12 MPa	Highly anisotropic	Highly biomimetic	High	Low	[45]
	SIS: 12–25 MPa					[46]
	Porcine meniscus: 110–200 MPa					[47,48]
ECM Components	250–500 kPa	Anisotropic	Biomimetic	High	Medium	[49]
Synthetic Polymers	200–5000 MPa	Highly anisotropic	Depends on the fabrication method	Low	High	[50]
Hydrogels	0.01–10 MPa	Isotropic	Depends on the fabrication method	Medium	High	[51]

**Table 2:** Common fabrication technologies used in meniscal tissue engineering.

Scaffold Structure	Fabrication Method	Pros & Cons	Reference
Sponge scaffold	Particulate leaching	(+) highly porous scaffolds with porosity values up to 93%	[70]
		(−) only used to produce thin membranes up to 3 mm thick	
	Gas foaming	(+) organic solvent-free process	[71,72]
		(−) a structure with largely unconnected pores	
		(−) non-porous external surface	
	Freeze drying	(+) highly porous scaffolds with porosity values >90%	[73]
		(+) reduction of toxic solvents use	
		(+) elimination of time-consuming drying and leaching processes of porogen components	
		(−) instability of the emulsion	
		(−) difficulty in controlling the pore size and porosity	
	Phase separation	(+) highly porous scaffolds with porosity values >90%	[74,75]
		(−) limited range of pore size (<200 um)	
		(−) difficult to control the micro- and macro-structure of the scaffold	
Non-woven fibrous scaffold	Electrospinning	(+) nanofibrous architectures	[79,80,81,82,83]
		(+) wide range of fiber diameters	
		(+) wide range of polymers can be used	
		(−) used solvents can be toxic	
		(−) limited capability to fabricate biomimetic structure	
Oriented/woven fibrous scaffold	FDM/PED	(+) layer by layer architecture	[84,85,86]
		(+) ability to fabricate complex structures	
		(−) low resolution	
		(−) limited range of materials	
	EHD-jetting	(+) layer-by-layer architecture	[68,87]
		(+) ability to fabricate complex structures	
		(−) used solvents can be toxic	
